# Implementation of the Enhanced Moderated Online Social Therapy (MOST+) Model Within a National Youth E-Mental Health Service (eheadspace): Protocol for a Single Group Pilot Study for Help-Seeking Young People

**DOI:** 10.2196/resprot.8813

**Published:** 2018-02-22

**Authors:** Simon Rice, John Gleeson, Steven Leicester, Sarah Bendall, Simon D'Alfonso, Tamsyn Gilbertson, Eoin Killackey, Alexandra Parker, Reeva Lederman, Greg Wadley, Olga Santesteban-Echarri, Ingrid Pryor, Daveena Mawren, Aswin Ratheesh, Mario Alvarez-Jimenez

**Affiliations:** ^1^ Orygen, The National Centre of Excellence in Youth Mental Health Parkville Australia; ^2^ Centre for Youth Mental Health The University of Melbourne Melbourne Australia; ^3^ Orygen Youth Health Northwestern Mental Health Melbourne Australia; ^4^ School of Psychology Australian Catholic University Melbourne Australia; ^5^ headspace National Youth Mental Health Foundation Melbourne Australia; ^6^ School of Computing and Information Systems The University of Melbourne Melbourne Australia; ^7^ Institute of Sport, Exercise and Active Living Victoria University Melbourne Australia; ^8^ Universitat Rovira i Virgili Tarragona Spain

**Keywords:** internet, social networking, young adult, adolescent, mental heath

## Abstract

**Background:**

There is a substantial need for youth electronic mental health (e-mental health) services. In addressing this need, our team has developed a novel moderated online social therapy intervention called enhanced moderated online social therapy (MOST+). MOST+ integrates real-time, clinician-delivered Web chat counseling, interactive user-directed online therapy, expert and peer moderation, and private and secure peer-to-peer social networking. MOST+ has been designed to give young people immediate, 24-hour access to anonymous, evidence-based, and short-term mental health care.

**Objective:**

The primary aims of this pilot study were to determine the feasibility, acceptability, and safety of the intervention. Secondary aims were to assess prepost changes in key psychosocial outcomes and collect qualitative data for future intervention refinement.

**Methods:**

MOST+ will be embedded within eheadspace, an Australian youth e-mental health service, and will be evaluated via an uncontrolled single-group study. Approximately 250 help-seeking young people (16-25 years) will be progressively recruited to the intervention from the eheadspace home page over the first 4 weeks of an 8-week intervention period. All participants will have access to evidence-based therapeutic content and integrated Web chat counseling. Additional access to moderated peer-to-peer social networking will be granted to individuals for whom it is deemed safe and appropriate, through a three-tiered screening process. Participants will be enrolled in the MOST+ intervention for 1 week, with the option to renew their enrollment across the duration of the pilot. Participants will complete a survey at enrollment to assess psychological well-being and other mental health outcomes. Additional assessment will occur following account deactivation (ie, after participant has opted not to renew their enrollment, or at trial conclusion) and will include an online survey and telephone interview assessing psychological well-being and experience of using MOST+.

**Results:**

Recruitment for the study commenced in October 2017. We expect to have initial results in March 2018, with more detailed qualitative and quantitative analyses to follow.

**Conclusions:**

This is the first Australia-wide research trial to pilot an online social media platform merging real-time clinical support, expert and peer moderation, interactive online therapy, and peer-to-peer social networking. The importance of the project stems from the need to develop innovative new models for the efficient delivery of responsive evidence-based online support to help-seeking young people. If successful, this research stands to complement and enhance e-mental health services in Australia.

## Introduction

Relative to the burden of disease, engagement rates with mental health services fall disappointingly below those for physical ill health [1]. This is especially true for young people aged 16 to 25 years—a period known for the peak onset of high prevalence and severe mental disorders such as depression and anxiety [2]. Given young people’s enthusiasm for Web-based communication, the development of innovative, online psychosocial interventions may assist to improve treatment acceptability and access for young people experiencing mental ill health [3]. Furthermore, social networking sites are rapidly becoming an essential avenue for social communication and support [4] and are likely to be pivotal to youth engagement with mental health services [5].

Mobile and digital technologies are developing at a phenomenal rate and hold great promise for influencing and transforming treatment delivery for emerging mental health conditions [6]. These technologies can be particularly useful for young people, as individuals under 25 years are by far the greatest users of internet resources [7]. For instance, over 95% of young Australians use the internet daily, and 97% have access to mobile phones, most of which are internet-enabled [8].

Although young people tend to nominate face-to-face support as their preferred mode of help-seeking for depression [9], a significant number indicate preference for online intervention because of the added anonymity and immediacy associated with the online environment [10]. Given their ability to transcend geographical boundaries and provide 24-hour accessibility, online interventions have the added potential to reach young people who may not be able or inclined to seek help from traditional sources [11,12].

In recent years, a range of online interventions have been successfully trialed for the management of a number of mental disorders, with research supporting the efficacy of these interventions in alleviating anxiety and depressive symptoms [13-15]. Online interventions have been reported as being as effective as face-to-face therapy [11], and a number of countries now recommend the use of online interventions within clinical guidelines for the treatment of high prevalence mental disorders such as depression [16].

At present, electronic mental health (e-mental health) services provide either nonclinical peer-to-peer support or clinical support or online therapy with no integrated online social networking and peer support or moderation [17-19]. In 2011, Australia’s headspace—National Youth Mental Health Foundation (headspace) began providing an e-mental health service (eheadspace) to extend the enhanced primary care services that had been provided through headspace centers across Australia since 2006 [12]. The headspace centers provide youth-friendly and low-stigma access to mental health care and have been effective in improving the mental health outcomes of those who access their services [20]. The eheadspace service delivers online chat, email, and telephone counseling provided by qualified clinicians at no cost to the young person in an effort to support young Australians who are unable or disinclined to seek professional support in person [12]. The eheadspace service is very popular among young Australians with over 25,000 clinical contacts per year across the country. Given the high demand and acceptability of this service, the next generation of eheadspace could harness the potential of Web 2.0 by fully integrating online professional support with high-quality, engaging, user-directed therapy and peer-to-peer social networking capability. These innovations have the potential to cater for the needs and preferences of more young people and provide a 24/7 therapeutic environment that extends beyond 1:1 clinical support to enable its scalability.

We have pioneered a new model of online behavioral interventions entitled *Moderated Online Social Therapy* (MOST) [21-27]. The MOST model integrates (1) Peer-to-peer online social networking, (2) Individually tailored interactive psychosocial interventions focused on using and developing self-identified strengths, and (3) Involvement of expert mental health and peer moderators to ensure the safety of the intervention. This model has been successfully piloted with young people experiencing first episode psychosis (the *Horyzons* study [21]) and major depression (the *Rebound* study [25,27]), as well as with carers of young people recovering from a first episode of psychosis (the *Altitudes* study [23]) and diagnosed with anxiety or depression (the *Meridian* study [24]).

To meet the specific needs of young people accessing eheadspace services, the MOST model has been upgraded to integrate Web chat services provided by eheadspace within a secure online social media environment. Referred to as MOST+, this enhanced moderated online social therapy model offers short-term interventions that capitalize on anonymity, social networking, and the broad availability and comparative low cost of social media–based interventions. By supplementing Web chat and telephone services with a wider array of online support, MOST+ stands to offer young people access to multiple modes of therapy, catering to the needs of different individuals.

Primary aims of the MOST+ pilot (Trial Registration: ACTRN12617000370303) were to determine the feasibility, acceptability, and safety of the intervention for young people aged 16 to 25 years who have accessed eheadspace services for mental health support. A secondary aim of the project was to assess changes in key psychosocial outcomes (ie, psychological distress, functional impairment, satisfaction with life, mental well-being, social support and isolation, and strengths use and knowledge) from the point of engagement to post intervention.

## Methods

### Study Design and Setting

In this single-group prepost pilot, the MOST+ intervention is embedded within existing eheadspace services. eheadspace is a national, federally funded e-mental health service for young Australians. eheadspace provides a youth-friendly, confidential, and free internet-based mental health support and information service. The eheadspace service is professionally staffed by qualified and supervised clinicians offering synchronous Web chat, email support, and telephone-based mental health intervention to young people aged 12 to 25 years Australia-wide. Clinicians working within the MOST+ platform will be based at the eheadspace operation center. They will be allied health professionals (eg, clinical and generalist psychologists, social workers, occupational therapists, and mental health nurses) who have specialist training and experience in the delivery of e-mental health support to young people in distress. Approximately 20 eheadspace clinicians will be trained for the MOST+ study, with two or three clinicians rostered on to provide support via MOST+ per day.

### Ethics, Consent, and Permissions

Research ethics approval for the MOST+ pilot was provided by the University of Melbourne Human Research Ethics Committee; Ethics ID: 1545798. All participants will be required to provide informed online consent.

### Participants

Inclusion criteria for participants will reflect the real world clinical characteristics of young people accessing e-mental health support, that is, (1) Help-seeking young people with concerns about their own mental health, (2) Age of 16 to 25 years inclusive, and (3) Ability to give informed consent and comply with study procedures. By the age of 16 years, young people in Australia are considered able to make independent decisions regarding their health care [28]. An age range of 16 to 25 years reflects the agency of young people to make their own health service choices, while ensuring the age appropriateness of the therapeutic material and social networking features of the MOST+ platform. Existing eheadspace clients meeting these criteria will be eligible to use the MOST+ intervention.

**Figure 1:**
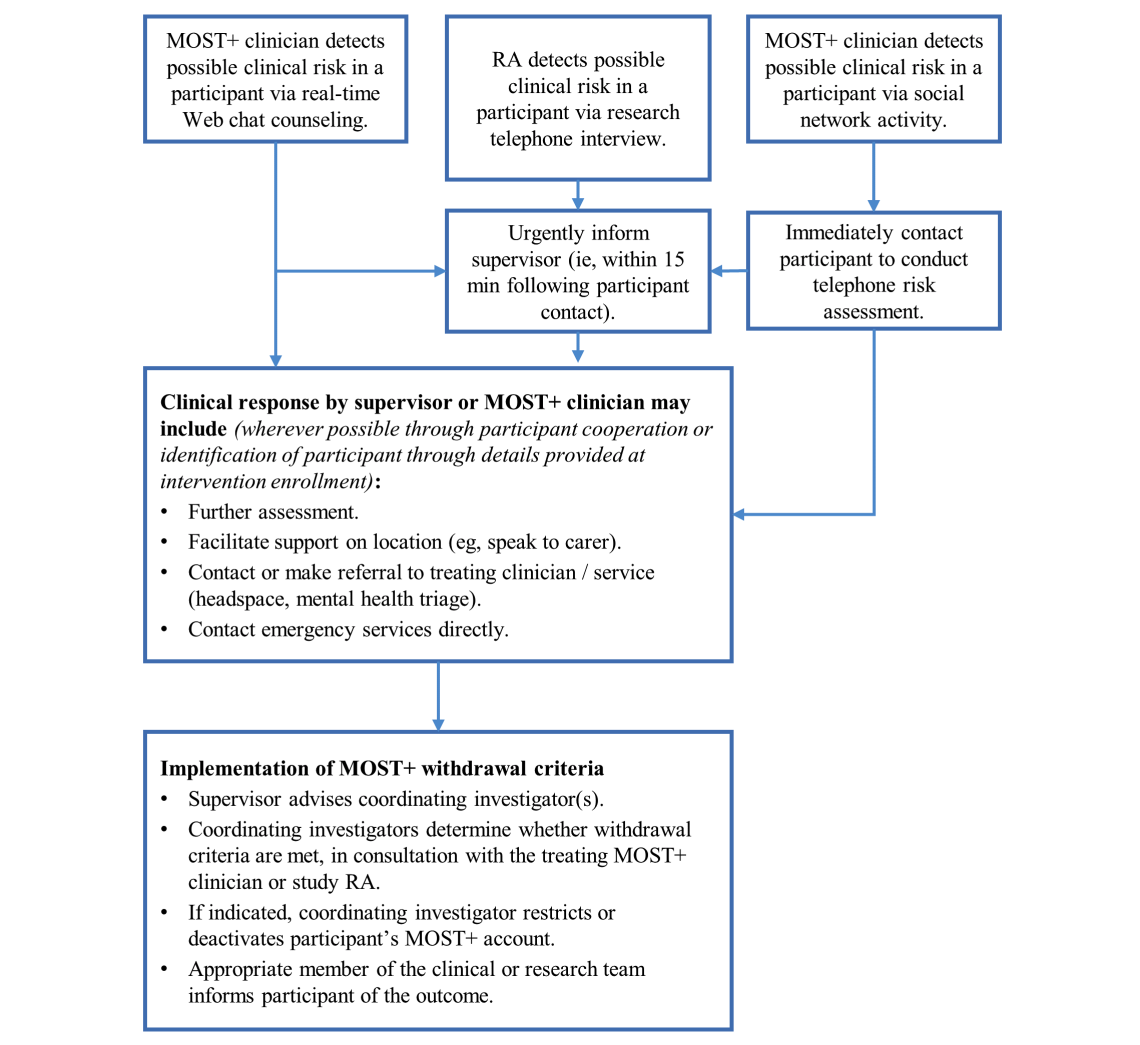
Enhanced moderated online social therapy (MOST+) intervention safety algorithm. RA: research assistant.

Consistent with current eheadspace practice, participants will not be excluded from the study on the basis of clinical characteristics. However, the following exclusions will apply for access to the social networking component of the intervention: (1) acute risk of self-harm requiring urgent intervention (ie, suicidal ideation with a current plan and intent to enact this plan) indicated by a young person in Web chat or by endorsing screening questions 7 and 8 in the registration survey; (2) an eheadspace clinician, in consultation with a supervisor, deems participation in the MOST+ social network likely to interfere with appropriate clinical management of mental health symptoms (eg, psychosis) or increase risk of harm to self or others (see Figure 1); and (3) inability to confirm age and conduct induction via research assistant (RA) telephone contact.

### Recruitment

Participants will be progressively recruited into the study, via an opt-in process, at point of entry to eheadspace through a link on the home page [29]. This link will be active during times when eheadspace clinicians are available on the MOST+ platform (ie, 4 PM to midnight Australia Eastern Daylight Time). Given high monthly eheadspace Web chat traffic (ie, >1000 new users aged 16-25 years), a recruitment period of approximately 4 weeks is considered adequate to ensure that the target of 250 participants is achieved. The availability of MOST+ will be highlighted on the eheadspace home page throughout the recruitment period. Study information will indicate beneficial features associated with the intervention (ie, instant access to specifically designed therapy content, potential access to social or emotional support from other help-seeking young people, clinicians, and peer moderators via the social network).

### Intervention

Participants will use MOST+, an interactive, purpose built online platform designed to deliver responsive evidence-based support to help-seeking young people. The intervention is designed to better meet the increasing demand for youth e-mental health support and is not intended as a replacement for recommended treatments for ongoing mental illness.

#### Accessing Enhanced Moderated Online Social Therapy

**Figure 2:**
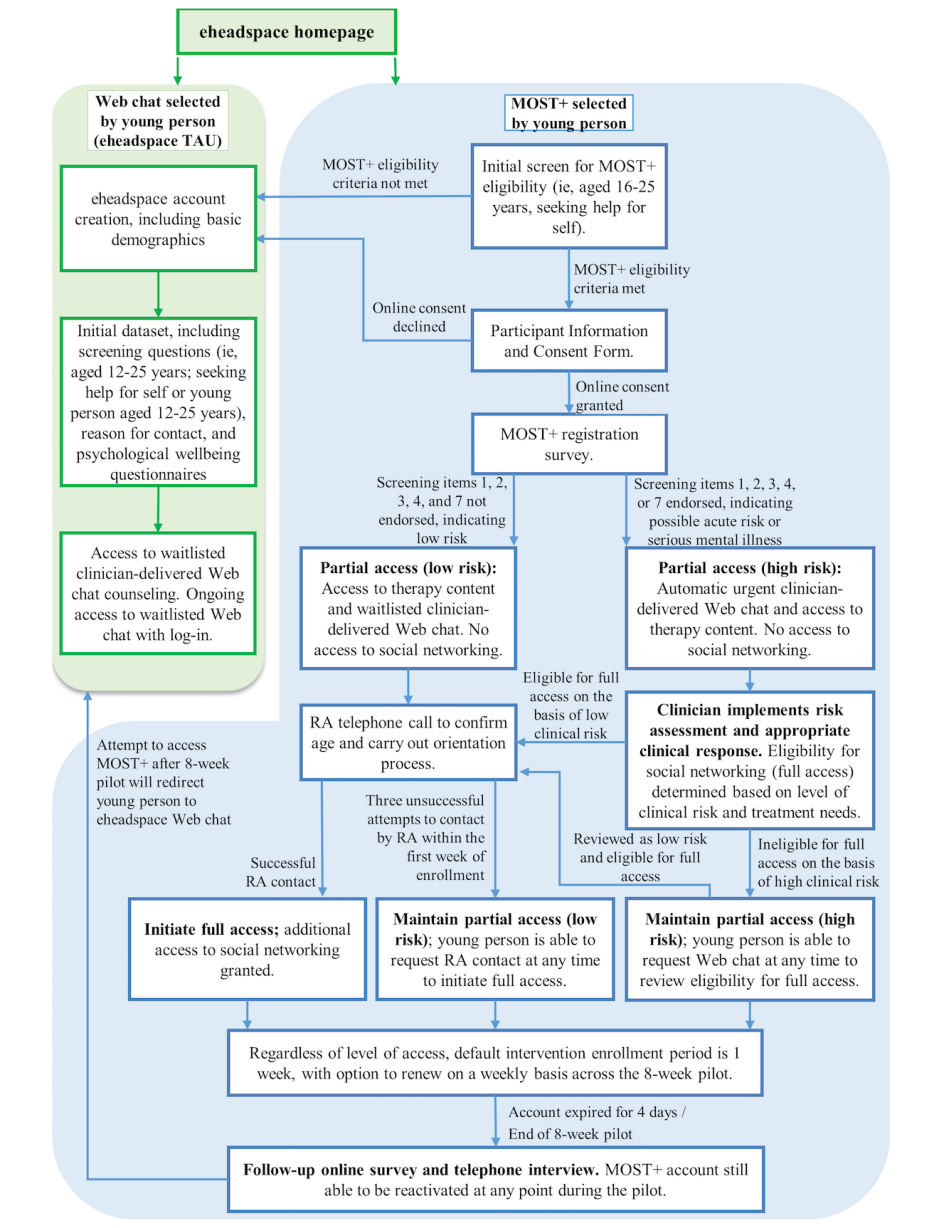
Study procedure. MOST+: enhanced moderated online social therapy; RA: research assistant; TAU: treatment as usual.

Only young people who indicate online that they understand and consent to study procedures will continue to the online MOST+ registration survey. This survey will assess outcomes at baseline and will consist of items administered within the registration process for standard eheadspace services, with additional questions included to assess all outcomes (see Table 1). This will take approximately 10 min to complete. Following this survey, all users will be granted access to real-time, clinician-delivered Web chat and user-directed psychosocial interventions (ie, partial access). Additional access to moderated peer-to-peer social networking (ie, full access) will be granted based on a three-tiered screening process designed to determine the safety and appropriateness of this component of the intervention for each user. Further details of the procedures for intervention implementation are provided in Figure 2.

#### Partial Access and Strengths

Partial access will comprise access to clinician-delivered Web chat counseling between 4 PM and midnight Australia Eastern Standard Time and 24/7 access to interactive psychosocial interventions. MOST+ adopts a strengths-based approach [45] through which users are guided and prompted to identify, discuss, and exercise key personal strengths within the online social environment and in real life to foster positive mood, enhance social connectedness and self-efficacy, and build resilience. This approach is based on the novel positive psychology model which proposes that psychosocial interventions should aim to build strengths, meaning, and purpose, as well as relieve symptoms. Participants will be prompted to choose their top five strengths from a list of 24 on the MOST+ platform. Each strength is presented with an image and a short description to help participants choose those which apply to them best.

#### Therapy Steps

Psychosocial interventions in MOST+ take the form of brief therapy comics, called “Steps,” designed to guide the user through various situations and address salient concerns for the broad population of young people seeking eheadspace services (eg, managing immediate distress, identifying depression and anxiety, accessing offline social and professional support, substance abuse, and vocational choices). Participants will also have access to short descriptions of specific actions, called “Do its,” which can be completed to practice using their personal strengths. Therapeutic content in MOST+ is informed by evidence-based psychosocial interventions developed by our group using participatory design principles [46,47]. In MOST+, content has been adapted to address salient concerns for the broad population of young people seeking eheadspace services.

**Table 1:** Schedule of assessments.

Measures		Assessment time point	
		Baseline	Follow-up
**Self-report assessments**			
	Demographics questionnaire^a^	✓	
	Internet usage	✓	
	Presenting problems^b^	✓	
	Kessler Psychological Distress Scale (K10)^a^ [30,31]	✓	✓
	Functional impairment^a^	✓	✓
	3 items from the Warwick-Edinburgh Mental Wellbeing Scale^a^ [32]	✓	✓
	Satisfaction with Life Scale^a^ [33,34]	✓	✓
	UCLA Loneliness Scale [35,36]	✓	✓
	Friendship Scale [37]	✓	✓
	Strengths Use and Knowledge Scale [38,39]	✓	✓
	Patient Health Questionnaire-9 [40] (depression)	✓	
	Freiburg Mindfulness Inventory [41]	✓	✓
	Perceived Stress Scale [42]	✓	✓
	Basic Psychological Need Satisfaction Scale [43,44]	✓	✓
**User experience assessments**			
	MOST+^c^ usability questionnaire		✓
	Semistructured phone interview		✓
	Online usage monitoring	Throughout trial	Throughout trial

^a^Assessment items included in the eheadspace Minimum Data Set (MDS).

^b^Assessed using six eheadspace MDS items and one additional item used to identify users who require urgent clinician-delivered Web chat on the basis of possible clinical risk.

^c^MOST+: enhanced moderated online social therapy.

#### Social Networking

In addition to this content, participants with full access will be able to communicate with other users and peer moderators in “The Café” and “Talk It Out” sections of the MOST+ platform. The “cafe” includes a Web feed (or news feed) where users and moderators can create posts to share thoughts, information, pictures, and videos and respond to other users’ posts by commenting or “liking” content. The system includes a “network” (similar to a “friends” function on Facebook) that displays personalized profiles for all active members. Users can also communicate in a collaborative problem-solving forum called “Talk It Out.” Participants can suggest topics and discuss solutions with moderators and other young people. Moderators will encourage users to define the problem, brainstorm possible solutions, identify pros and cons, and summarize possible choices. This function uses an evidence-based, problem-solving framework and has been piloted successfully within the MOST model [21].

#### Duration of Access

**Figure 3:**
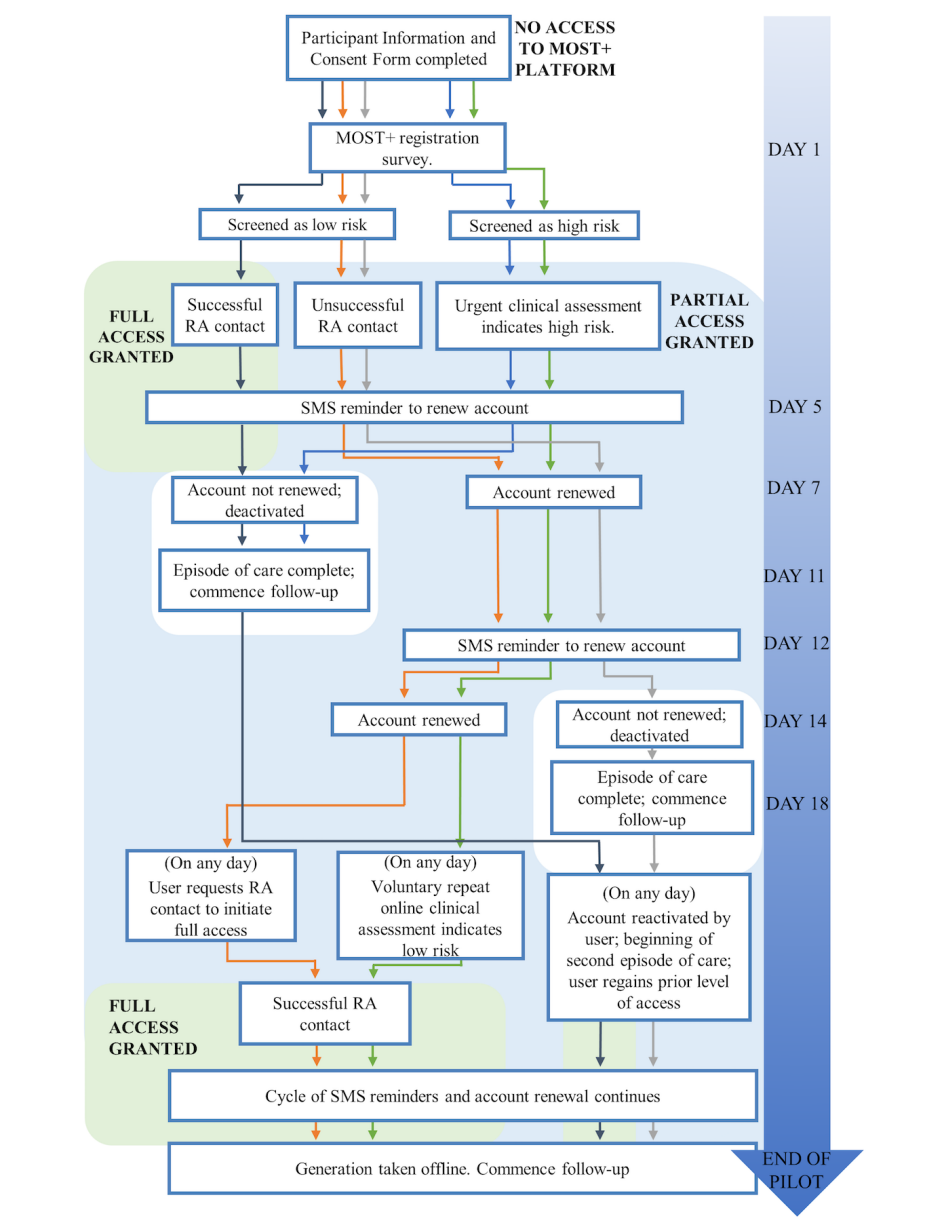
Example participant timelines through the enhanced moderated online social therapy (MOST+) intervention. RA: research assistant; SMS: short message service.

Regardless of level of access, all participants will be enrolled in the MOST+ intervention for 1 week, with the option to extend their enrollment on a weekly basis across the study intervention period. Thus, participants’ period of enrollment in the intervention will range from a minimum of 1 week to a maximum of 8 weeks. Participants will be able to reactivate an expired account at any time during the study intervention period, and this will be treated as a second episode of care. Following the conclusion of the pilot study, participants will be redirected to eheadspace if they attempt to log into the MOST+ platform. See Figure 3 for examples of participants’ possible enrollment time lines, with each colored line representing a different potential time line over the duration of the pilot.

#### Acute Risk

Any signs of possible acute risk or inappropriate use of the MOST+ system will be discussed with the principal investigators (PIs). eheadspace clinical staff, in consultation with the MOST+ coordinating investigators, will decide whether to withdraw participants from the pilot, and this will be communicated to the participant by the most appropriate member of the clinical or research team. A return to partial access, with close monitoring by the MOST+ clinicians, will be implemented in preference to full withdrawal wherever possible.

#### Integration With eheadspace

eheadspace Web chat will be fully integrated with evidence-based therapeutic content and social networking features within MOST+. Clinicians will facilitate real-time Web chat sessions via a function within the MOST+ platform. eheadspace Web chat involves clinician-delivered online counseling in real time and is focused on reducing immediate distress, supporting positive self-care, and facilitating referral to additional supports where appropriate. Web chat may also include therapeutic interventions targeting high prevalence mental health conditions and coordinated care responses for complex and high risk clients. Wherever appropriate at the conclusion of a Web chat session, MOST+ clinicians will suggest that users complete specific, relevant content from within the online platform based on the context of their chat session. This will be bolstered outside of the live Web chat sessions by the clinicians, who will engage active users with therapeutic content by posting links within the social network, or via messaging for those participants without access to peer-to-peer social networking.

### Moderation

The MOST+ social network incorporates two types of moderation: expert moderation by eheadspace clinicians and peer moderators. Moderation will follow a manualized, theory-driven model (ie, the supported accountability model), which has been successfully utilized in previous MOST studies [21,25] to encourage use of the platform through participants’ sense of accountability to a trustworthy and experienced online “coach” [48]. MOST+ peer moderation has been informed by social cognitive theory and recent evidence from the computing and information systems field, which posit that peer moderators can model appropriate online behaviors through demonstrating desired actions and behaviors [49].

MOST+ clinicians will monitor new contributions to the network for indicators of clinical risk. The social network will be moderated by an on-duty MOST+ clinician daily. Safety checks will be undertaken during weekdays and also on weekends. MOST+ clinicians will receive initial training from experienced clinicians from the research team, and will be in close contact throughout the trial. Clinicians will receive regular supervision throughout the trial from their usual eheadspace shift supervisors.

Peer moderation will be conducted by “super users,” who will be active members of the MOST+ social network, providing guidance and peer-to-peer support and fostering hope and empowerment [50]. Their role will include welcoming new members of the social network and modeling positive engagement with the system. Super users will post content and interact with other users in The Café and guide problem-solving discussions in Talk It Out. Individuals eligible to become super users will be aged 16 to 25 years and known to be in the later stages of recovery following a recent lived experience of mental ill health, as indicated by the youth participation coordinator within Orygen, The National Centre of Excellence in Youth Mental Health. A precondition of super users’ involvement is the development of a plan indicating early warning signs. For example, a super user may identify patterns of irritability, withdrawal, and sleep disturbance as early warning signs for more significant distress.

Super users are trained in how to use the MOST+ platform and employed to moderate discussions and help participants use the site. Eligible young people will be selected to become super users if they can demonstrate via an interview process that they have (1) Comprehensive self-care strategies, (2) Nominated support services or people, and (3) Self-awareness of early warning signs indicating possible wellness fluctuation. The interview process will be facilitated by an experienced youth participation coordinator at Orygen and will provide an opportunity to ensure that all selected super users have a clear understanding of their role and are eager and able to meet the time commitments involved. Consistent with the Orygen Youth Health policy for peer support workers, MOST+ super users will be treated as “reimbursed volunteers” and compensated AUS $30 per hour for training, supervision meetings, and moderation shifts.

Moderation integrity will be ensured through a detailed moderation manual. The research team will regularly review recorded Web chat sessions and contributions to the MOST+ social network to assess clinician adherence to the moderation manual and identify areas for discussion within supervision meetings.

Supervision with MOST+ peer moderators and clinicians during the course of the pilot will provide ongoing opportunities to receive feedback and collaboratively identify opportunities for further improvements to the intervention. At the conclusion of the pilot, all MOST+ clinicians and peer moderators will be invited to attend an audio-recorded focus group or individual interview that will be aimed at gaining detailed feedback on their experience of providing support within the MOST+ platform. Information gained through this process will indicate the acceptability of MOST+ from moderators’ perspectives and be used to further refine the intervention and moderator training resources before any future implementation of MOST+.

### User Anonymity and Privacy

The MOST+ platform has been designed to maximize user privacy and choice within the social network. Participants are able control their level of identification within the network (ie, first name and/or photo, or complete anonymity). Should a participant become concerned about their privacy during their course of participation, they are able to “switch off” their profile to hide all of their past activity within “The Café” and anonymize their contributions to “Talk It Outs.” With the exception of the research and clinical team, user contributions to the network are viewable by current members of the network only. In addition, user activity (eg, posts and profile information) is not permanent within the network. That is, users’ accounts are renewed on a weekly basis through a user driven “opt-in” system, and all user activity is automatically hidden from the social network for those who choose not to renew their account. Although users are able to reactivate their account and regain their profile information at any point, all other user activity is permanently removed following a 4-day grace period during which users can reactivate their account. Users will be made aware that any user activity information that disappears from the social network is retained by the researchers for the purpose of analysis.

### Outcomes

The primary outcome variables will be intervention feasibility, acceptability, and safety. Assessment of intervention feasibility will involve reviewing a log of participants' access to, and usage of, the MOST+ platform. Access data will include the number of participants recruited and retained through the study, participants’ level of access, reasons for maintained partial access following initial intervention enrollment, and details of any full or partial withdrawals from the intervention. Usage data will include the number of log-ins, steps completed, and contributions to Talk It Out and The Café. This is the first study piloting an intervention of this nature and scale for help-seeking young people and, as such, registration and usage rates are difficult to predict. For this reason, an *a priori* criterion for the number of active users that would indicate study feasibility has not been defined.

Acceptability will be assessed at follow-up using specially designed questions administered within a semistructured feedback interview. Acceptability will be considered achieved if (1) Participants provide ratings of the MOST+ platform averaging above three out of five across feedback questions regarding ease of use, relevancy, helpfulness, and overall experience; (2) At least 60% of participants report that the MOST+ intervention provided timely, relevant, and helpful support at semistructured follow-up interview; and (3) At least 80% of respondents would recommend MOST+ to other young people experiencing difficulties.

The pilot will be considered to indicate safety of the MOST+ intervention if (1) At least 90% of participants report the online intervention to be safe via semistructured feedback interview at follow-up; (2) None of the participants experience a serious adverse event as a result of their engagement with the system during their intervention period (ie, none of the participants experience a significant deterioration in mental health or self-harm in response to intervention content, as reported by the participant or determined by treating health care professionals); and (3) There are no unlawful entries into the MOST+ system detected by study programmer during the 8-week pilot.

As shown in Table 1, all secondary outcomes will be assessed at both baseline and follow-up, with additional MOST+ user experience items assessed at follow-up. Secondary outcome variables will include self-report measures of perceived stress, psychological distress, functional impairment, mental well-being, satisfaction with life, social support and isolation, strengths use, depression, mindfulness, and components of self-determination theory. These outcomes have been chosen as they are relevant to young people’s perceptions of well-being and quality of life and may be amenable to change via the intervention. Strengths use represents a key target of MOST+ therapeutic content and will be investigated as a potential mediator of pre- to postintervention changes to other assessed indicators of psychosocial well-being. The registration survey will also include questions assessing frequency of internet use.

A number of additional quantitative and qualitative intervention feedback items will be administered within a semistructured telephone interview format at follow-up to gain a detailed understanding of areas for improvement within the system. The interview questions are based on the user experience approach as described by Bargas-Avila and Hornbaek [51]. Interviews will allow the study RA to explore six themes: overall impressions of MOST+, patterns of system use, feedback on specific aspects (eg, psychosocial interventions), Web chat and moderation, interactions with other MOST+ users (if relevant), and suggestions for system improvements.

### Sample Size

The majority of young people who access eheadspace make use of the service on one or two occasions only, and a primary objective of MOST+ is to facilitate responsive and effective intervention within this short window. To mirror likely real-world implementation of the MOST+ model, we will also be recruiting young people to the online intervention regardless of whether they can be contacted via telephone within the first week of study enrollment (see Figure 2). Although follow-up periods will be tailored to participants’ initial period of intervention engagement, it is likely to be challenging to retain participants who have engaged only briefly and/or were unable to be contacted via telephone at enrollment. A sample size of approximately 250 participants will therefore be necessary both to provide an adequate buffer against attrition during the study and to ensure that there is a sufficient number of active users of MOST+ for the social networking aspect of the intervention to function effectively throughout the 8-week pilot.

### Data Collection and Management

All participants will receive one AUS $30 voucher as reimbursement for their time in completing registration and another for completing follow-up. Follow-up assessment will occur approximately 4 days after initial account deactivation (ie, 4 days after a user has opted not to renew their account for an additional week). For those participants who maintain active enrollment across the intervention period, follow-up will occur as soon as possible following conclusion of the pilot. The exact duration of follow-up cannot be estimated as the number of participants enrolled in MOST+ will not be capped. To minimize attrition, participants will receive a short service message notification that their online follow-up survey is due and will be able to complete survey items either online or via telephone. Participants who do not complete the follow-up survey will be reminded via phone call by an RA. Both baseline and follow-up assessments will be designed to minimize participant burden, while ensuring that requirements for the eheadspace Minimum Data Set (MDS; ie, routine data about each occasion of service) are met.

The MOST+ system and data generated through participants’ use of the online platform will be hosted on a secure University of Melbourne Web server. Data collected via online surveys and telephone interviews will be entered electronically and also centrally stored on a University of Melbourne Web server. At the conclusion of the study, this data will be exported and stored securely in a password protected file on the server at Orygen. In addition, data collected as part of eheadspace MDS requirements will be provided to headspace at the conclusion of the study. For each MOST+ account, clinical content collected via Web chat and surveys will be copied into an eheadspace account to ensure continuity of care should the participant continue using eheadspace services post trial.

All of the PIs will have access to the final pilot dataset for the purpose of characterizing the participant sample, characterizing the activity within the MOST+ system, and testing the study hypotheses. The study RAs will also have access to the dataset for the purpose of data entry and management. Expert moderators and eheadspace clinicians working within MOST+ will have access to individual and aggregated usage data available within the moderator interface.

Given the relatively small scale, brief duration and primary aims of assessing the feasibility, acceptability, and safety of the intervention, an independent data safety monitoring committee will not be established for this study.

### Data Analysis

Frequency, duration, and patterns of use of MOST+ will be tracked in real time. Up-to-date charts will be visible on demand to MOST+ staff via the moderator interface of the online platform. This data will be aggregated into simple descriptive statistics to characterize participants’ use of the intervention. In addition, overall rates of MOST+ participation and aggregated data from the user feedback questionnaire will be compared with the *a priori* acceptability and safety criteria to determine success of the pilot. Supplementary paired samples *t* tests will be conducted to assess pre- to postintervention changes to outcomes. System usage will be examined as a covariate.

### Harms

System and privacy protection will be monitored by the study programmers. All attempts, both successful and unsuccessful, to log into the site are recorded in a database table that can be monitored. This table also informs a security mechanism whereby if five incorrect log-in attempts are made from the same internet protocol (IP) address within a 1-hour period, then an email alerting the development team is sent, and log-in access to the site is blocked for that IP address for the next hour. Online safety will be monitored proactively by the MOST+ clinicians, with supervision from the senior researchers. MOST+ clinicians will have the authority to respond to users’ reports of inappropriate material within the online platform and to automated email reports of posts that include potentially offending material. eheadspace staff will respond to indications of clinical risk according to the MOST+ safety algorithm (see Figure 1).

An important function of eheadspace and MOST+ is to facilitate support, containment, and appropriate referral wherever possible in instances of acute clinical risk. Successful implementation of the MOST+ clinical safety protocol will therefore be treated as appropriate use of the intervention and will not be automatically recorded as an adverse event. All activations of the safety protocol will nevertheless be recorded and reviewed by the coordinating investigators as part of ongoing monitoring of the feasibility and safety of the intervention. In addition, serious adverse events will be monitored, recorded, and reported to the study sponsor and ethics committee in accordance with Good Clinical Practice guidelines. Serious adverse events will be tracked throughout the duration of the study. Serious adverse events are defined as events that (1) Result in death, (2) Are life-threatening, (3) Require inpatient hospitalization or prolongation of existing hospitalization, (4) Result in persistent or significant disability or incapacity, or (5) Are other medically important events or reactions.

For each moderate to high-risk case, eheadspace documentation includes: (1) The facts of the case; (2) A clear opinion as to the level of risk; (3) A rationale for this opinion; (4) A statement of any consultations with carers and other involved agencies; and (5) Completion of a care plan. This reporting process will be followed by all eheadspace clinicians working within the MOST+ interface, and case notes involving activation of the MOST+ clinical safety protocol will be reviewed by the coordinating investigators.

In the event that clinical deterioration in a research participant is evident and attributable to usage of the MOST+ system, the relevant MOST+ clinician will communicate with the PIs. The investigators will subsequently inform the ethics committee. In addition, the coordinating investigators will inform the ethics committee in the unlikely event of a security breach of the online platform which compromises the privacy of participant information. The occurrence of either of these two events will be taken to indicate an unacceptable safety risk of the MOST+ system, as per the *a priori* intervention safety criteria.

## Results

Recruitment for the study commenced in October 2017. We expect to have initial results in March 2018, with more detailed qualitative and quantitative analyses to follow.

## Discussion

The aim of this project was to pilot test the next generation of eheadspace services. As such, MOST+ has been designed to harness the popularity and high demand of eheadspace by integrating a state-of-the-art online social media–based platform that weaves together attractive evidence-based, therapeutic content; peer moderation; peer-to-peer online social networking; and one-to-one online clinical support. This novel platform has the potential to deliver an enriched 24/7 youth-led therapeutic environment that caters for the needs of more young people and increases the overall scalability of clinical e-mental health services.

To date, we have developed and implemented the MOST model for individuals, in particular clinical groups, who are already in contact with mental health services [21,25]. The current research will provide valuable insights into the implementation of MOST on a platform accessible to young people whose characteristics, including location, access to face-to-face health services, and mental health concerns, may vary considerably. The flexibility required for MOST+ to be suitable and useful for individuals with a range of concerns is built into the platform. Specifically, the use of user-driven social networking and problem solving and therapy content that focuses on users’ positive traits and well-being are intended to ensure the intervention is widely applicable. Furthermore, with this flexible, online model of care, MOST+ could be implemented across cultural and geographic boundaries.

By investigating feasibility, acceptability, and safety, the data from this pilot will indicate the suitability of progressing to a large randomized controlled trial to assess the clinical effectiveness and cost-effectiveness of this model. In addition, feedback from users, super users, and clinicians will inform the development of future MOST+ interventions, allowing for improvements to both content and implementation. As a first step in providing nation-wide access to integrated user-directed psychosocial interventions, peer-moderated social networking, and clinician chat, this pilot study stands to advance clinical e-mental health services in Australia.

The final trial dataset will be retained by Orygen, The National Centre of Excellence in Youth Mental Health.

## Abbreviations

e-mental health: electronic mental health
IP: Internet protocol
MDS: Minimum Data Set
MOST: moderated online social therapy
MOST+: enhanced moderated online social therapy
PI: principal investigator
RA: research assistant
SMS: short message service
TAU: treatment ass usual
